# 直径≤2 cm非小细胞肺癌淋巴结转移因素及采样范围评估

**DOI:** 10.3779/j.issn.1009-3419.2023.102.26

**Published:** 2023-07-20

**Authors:** Tianyu JIN, Zhicheng HE, Zhihua LI, Jianwei TANG, Jing XU, Weibing WU, Liang CHEN

**Affiliations:** 210000 南京，南京医科大学第一附属医院/江苏省人民医院胸外科; Department of Thoracic Surgery, the First Affiliated Hospital with Nanjing Medical University/Jiangsu Province Hospital, Nanjing 210000, China

**Keywords:** 肺肿瘤, 淋巴结转移, 危险因素, 肺叶特异性淋巴结清扫, Lung neoplasms, Lymph node metastasis, Risk factors, Lobe-specific lymph node sampling

## Abstract

**背景与目的** 越来越多的早期肺癌被及时诊断并手术治疗，但是系统性淋巴结清扫（systematic lymph node dissection, SND）不能为其带来足够的生存获益，甚至增加术后并发症发生概率。本研究旨在分析直径≤2 cm非小细胞肺癌（non-small cell lung cancer, NSCLC）淋巴结转移的危险因素以及不同肺叶好发的纵隔淋巴结转移站点，为手术提供参考意见。**方法** 纳入2009年12月至2019年12月于南京医科大学第一附属医院胸外科行肺叶切除术+淋巴结采样/清扫术治疗的肺结节患者（直径≤2 cm）共1051例，运用SPSS 26.0统计软件对资料进行统计分析，探讨淋巴结转移的危险因素以及不同肺叶好发的纵隔淋巴结转移站点。**结果** 1051例患者中发生淋巴结转移95例，转移率为9.04%，其中男性、病理非腺癌、肿瘤直径大于1 cm但不大于2 cm、存在气道播散（spread through air spaces, STAS）、胸膜侵犯（visceral pleural invasion, VPI）、脉管浸润、腺癌低分化、腺癌亚型为微乳头或实体型是淋巴结转移的危险因素（P<0.01）；男性、肿瘤直径大于1 cm但不大于2 cm、存在STAS、VPI、脉管浸润是淋巴结转移的独立危险因素（P<0.05）；右肺上、中叶易出现#2R、#4R、#9淋巴结转移（P<0.05），右肺下叶易出现#7淋巴结转移（P<0.05）；左肺上叶易出现#5、#6淋巴结转移（P<0.05），#7、#9转移无统计学差异（P>0.05）；N1组淋巴结与#2R、#4R、#5、#6、#7、#9组淋巴结转移有明显相关性（P<0.01）。**结论** 对于早期NSCLC，可以进行肺叶特异性淋巴结清扫（lobe-specific lymph node dissection, LSND），当患者为男性、病理为非腺癌、肿瘤直径大于1 cm但不大于2 cm、存在STAS、VPI、脉管浸润、腺癌低分化、腺癌亚型为微乳头或实体型时，其淋巴结转移的风险增高。

## Abstract (en)

**Background and objective** More early-stage non-small cell lung cancer (NSCLC) are diagnosed in time and treated surgically, but systematic lymph node dissection can not bring enough survival benefits for them, and even increase the probability of postoperative complications. This study aims to analyze the risk factors and evaluate mediastinal lymph node metastasis sites in different lung lobes for NSCLC with diameter ≤2 cm, so as to provide reference for surgery. **Methods** We collected 1051 patients with pulmonary nodule diameter ≤2 cm who were treated by pulmonary lobectomy with lymph node sampling/dissection in Department of Thoracic Surgery of the First Affiliated Hospital with Nanjing Medical University from December 2009 to December 2019. SPSS 26.0 statistical software was used for statistical analysis, to explore the risk factors and evaluate mediastinal lymph node metastasis sites in different lung lobes. **Results** 95 of 1051 (9.04%) patients presented lymph node metastasis. Male, pathological non-adenocarcinoma, 1 cm<tumor diameter≤2 cm, spread through air spaces (STAS), visceral pleural invasion (VPI), vascular invasion, low differentiation of adenocarcinoma, and micropapillary or solid adenocarcinoma subtype were the risk factors for lymph node metastasis (P<0.01). Male, 1 cm<tumor diameter≤2 cm, STAS, VPI and vascular invasion were independent risk factors for lymph node metastasis (P<0.05). Lymph node metastasis of #2R, #4R, #9 appeared in upper and middle lobe of right lung (P<0.05), and #7 appeared in right lower lobe (P<0.05). Lymph node metastasis of #5 and #6 in the left upper lobe was more common (P<0.05), while metastasis of #7 and #9 had no significant difference (P>0.05). Lymph nodes in group N1 were significantly correlated with lymph node metastasis in groups #2R, #4R, #5, #6, #7 and #9 (P<0.01). **Conclusion** Lobe-specific lymph node dissection (LSND) can be performed for early-stage NSCLC. Male, pathological non-adenocarcinoma, 1 cm<tumor diameter≤2 cm, STAS, VPI, vascular invasion, low differentiation of adenocarcinoma, and micropapillary or solid adenocarcinoma subtype would increase the risk of lymph node metastasis.

肺癌是中国常见的恶性肿瘤，由于吸烟人口的增加以及城市、农村工业化发展导致的空气质量下降，肺癌发病率正在逐年上升，其发病率仅次于乳腺癌，致死率位居第一^[1]^。肺癌主要分为小细胞肺癌和非小细胞肺癌（non-small cell lung cancer, NSCLC），其中NSCLC主要分为腺癌、鳞癌和其他组织类型，其中腺癌发病率最高。随着低剂量计算机断层扫描（low-dose computed tomography, LDCT）联合血清肺癌自身抗体的运用，更多早期肺癌被及时诊断^[2]^并获得治疗，提升了早期NSCLC患者的生存率。

肺癌的转移方式主要有淋巴结转移、血行转移、种植转移，其中NSCLC最为多发的转移方式是淋巴结转移。早期NSCLC患者的预后较好，但当其合并纵隔淋巴结转移时，患者预后较差，如在手术中未能及时地清扫转移淋巴结，将会错误地判断患者的分期，导致此类患者术后复发及转移的概率增加，因此选择合适的清扫淋巴结的方法，会提高早期NSCLC患者的总生存率以及无病生存率^[3]^。由于LDCT的普及，越来越多的以磨玻璃样结节（ground-glass opacity, GGO）为主的NSCLC得到了早期诊断，术前影像学诊断多为原位腺癌（adenocarcinoma in situ, AIS）、微浸润腺癌（minimally invasive adenocarcinoma, MIA）及浸润性腺癌（invasive adenocarcinoma cancer, IAC），根据2011年国际肺癌研究协会（International Association for the Study of Lung Cancer, IASLC）/美国胸外科学会（American Thoracic Society, ATS）/欧洲呼吸学会（European Respiratory Society, ERS）分类，浸润性腺癌又分为五种亚型^[4]^，对于不同影像学特征和病理亚型的NSCLC，术者常会选择不同的淋巴结清扫方式^[5]^。系统性淋巴结清扫（systematic lymph node dissection, SND）是进展期和局部晚期NSCLC的标准术式，但对于≤2 cm的早期NSCLC是否合适，仍存在争议^[6]^。有学者认为SND不能为无淋巴结转移的早期NSCLC患者提供足够的生存获益，反而会使术中风险增加并导致更多的术后并发症^[7]^。为减少上述事件的发生、应对患者进行个体化治疗，日本学者Okada等^[8]^首次提出了肺叶特异性淋巴结清扫（lobe-specific lymph node dissection, LSND）的概念，并且认为LSND在某些手术中可以替代SND，为患者带来相同的预后^[5]^。

本研究回顾性评估我院过去10年CT影像上直径≤2 cm的肺结节且行肺叶切除术+淋巴结采样/清扫术的NSCLC患者的淋巴结清扫情况，旨在探明淋巴结转移的相关风险因素以及不同肺叶的淋巴结采样/清扫区域，总结其相关规律。

## 1 资料与方法

### 1.1 一般资料

收集2009年12月至2019年12月间南京医科大学第一附属医院胸外科行外科手术治疗的直径≤2 cm的肺结节患者的资料，手术方式为全胸腔镜下肺叶切除术+淋巴结采样/清扫术。纳入标准：（1）患者手术资料详尽，术前行胸部CT平扫检查，且检查结果示结节最大直径≤2 cm；（2）患者行肺叶切除术，且淋巴结清扫方式为淋巴结采样/清扫术，N1、N2组淋巴结依据术前资料及术中所见，至少各采集一组；（3）既往无其他恶性肿瘤病史；（4）病理结果为NSCLC。排除标准：（1）患者手术资料缺失；（2）肺结节最大直径>2 cm；（3）5年内有其他恶性肿瘤病史；（4）病变位于双侧；（5）病理结果为小细胞肺癌或其他良性肿瘤。按上述标准共筛选出1051例患者，其中符合美国国立综合癌症网络（National Comprehensive Cancer Network, NCCN）指南淋巴结采集标准（采样肺门淋巴结+至少采样三组纵隔淋巴结）共485例。

### 1.2 术后病理分期

患者术后均进行常规病理检查，并由我科临床医师依据检查结果完善病理分期，研究中肿瘤原发灶-淋巴结-转移（tumor-node-metastasis, TNM）分期根据IASLC第八版肺癌分期标准，手术病理证实为原发性NSCLC。

### 1.3 统计学方法

数据均以SPSS 26.0统计软件分析，计数资料以率（%）表示，采用χ^2^检验或Fisher精确检验、多因素Logistic回归分析方法，其中淋巴结转移率=（淋巴结阳性例数/淋巴结采集例数）×100.00%，淋巴结清扫率=（该部位该组淋巴结采集例数/该部位肺叶切除术例数），P<0.05为差异具有统计学意义。

## 2 结果

### 2.1 1051例NSCLC患者的临床特征及淋巴结转移危险因素的χ^2^检验分析

纳入本次回顾性研究的1051例患者，手术方式均为肺叶切除术+淋巴结采样/清扫术，住院期间无死亡病例。淋巴结转移共95例，转移率为9.04%，N1淋巴结发生转移者34例，转移率为3.24%，N1、N2淋巴结共同发生转移者43例，转移率为4.09%，跳跃性N2淋巴结转移者18例，转移率为1.71%。统计1051例患者临床资料并行χ^2^检验分析发现，不同年龄、病变部位的患者淋巴结转移率比较无明显差异（P>0.05）；患者为男性、病理为非腺癌、肿瘤直径大于1 cm但不大于2 cm、存在气道播散（spread through air spaces, STAS）、胸膜侵犯（visceral pleural invasion, VPI）、脉管浸润、腺癌低分化、腺癌病理亚型为微乳头型或实体型是淋巴结转移的危险因素（P<0.01），见表1。

**表1:** 非小细胞肺癌淋巴结转移的危险因素

Clinical characteristics	Lymph node metastasis (+) (n=95)	Lymph node metastasis (-) (n=956)	χ^2^	P
Gender			18.944	<0.01
Male	56 (58.95%)	346 (36.19%)		
Female	39 (41.05%)	610 (63.81%)		
Age (yr)			0.158	0.691
<60	44 (45.8%)	418 (43.7%)		
≥60	51 (54.2%)	538 (56.3%)		
Tumor location			2.038	0.729
Right upper lobe	37 (38.95%)	392 (41.00%)		
Right middle lobe	7 (7.37%)	48 (5.02%)		
Right lower lobe	13 (13.68%)	152 (15.90%)		
Left upper lobe	21 (22.11%)	176 (18.41%)		
Left lower lobe	17 (17.89%)	188 (19.67%)		
Histology			7.267	0.026
Adenocarcinoma	85 (89.47%)	845 (88.39%)		
Squamous cell carcinoma	8 (8.42%)	39 (4.08%)		
Other	2 (2.11%)	72 (7.53%)		
Differentiation^a^			44.238	<0.01
Well	0 (0.00%)	43 (5.09%)		
Moderate	27 (31.76%)	432 (51.12%)		
Poor	33 (38.82%)	109 (12.90%)		
Unknown	25 (29.41%)	261 (30.89%)		
Pathological subtype^a^			38.393	<0.01
Lepidic/Acinous	19 (22.35%)	433 (51.24%)		
Papillary	26 (30.59%)	220 (26.04%)		
Micropapillary/Solid	28 (32.94%)	103 (12.19%)		
Unknown	12 (14.12%)	89 (10.53%)		
Tumor size			11.956	<0.01
≤1 cm	5 (5.26%)	188 (19.67%)		
1 cm<Diameter≤2 cm	90 (94.74%)	768 (80.33%)		
STAS			32.516	<0.01
Yes	12 (12.63%)	20 (2.09%)		
No	83 (87.33%)	936 (97.91%)		
VPI			16.570	<0.01
Yes	18 (18.95%)	67 (7.01%)		
No	77 (81.05%)	889 (92.99%)		
Vascular invasion			72.930	<0.01
Yes	15 (15.79%)	12 (1.26%)		
No	80 (84.21%)	944 (98.74%)		

^a^: Only the patients with invasive adenocarcinoma were emrolled for data analysis. 85 cases presented lymph node metastasis (+), and 845 cases presented lymph node metastasis (-). STAS: spread through air spaces; VPI: visceral pleural invasion.

### 2.2 淋巴结转移危险因素的Logistic回归分析

筛选出单因素分析有统计学意义的变量，对其进行多因素Logistic回归分析显示，患者为男性、肿瘤直径大于1 cm但不大于2 cm、存在STAS、VPI、脉管浸润是淋巴结转移的独立危险因素（P<0.05），见表2。

**表2:** 淋巴结转移患者多元Logistic回归分析

Clinical characteristics	SEM^a^	Wald	OR^b^	P
Gender (male)	0.233	12.731	2.297 (1.455-3.628)	<0.01
Tumor size (1 cm<Diameter≤2 cm)	0.475	8.216	3.905 (1.528-9.912)	<0.01
STAS	0.471	4.091	2.592 (1.030-6.526)	0.043
VPI	0.455	24.806	9.630 (3.950-23.482)	<0.01
Vascular invasion	0.316	8.336	2.490 (1.340-4.624)	<0.01

^a^: standard error of mean; ^b^: odds ratio.

### 2.3 不同部位肺叶切除术的淋巴结采样/清扫率及转移情况

不同部位肺叶切除术淋巴结采样/清扫率超过50.00%的分别为：（1）右肺上叶、右肺中叶（484例）：#2R、#4R（96.9%）、#7（92.36%）、#10（90.50%）、#11（66.32%）；（2）右肺下叶（165例）：#2R、#4R（80.61%）、#7（96.36%）、#9（55.15%）、#10（78.18%）、#11（84.85%）；（3）左肺上叶（197例）：#5、#6（96.45%）、#7（90.36%）、#9（65.99%）、#10（85.79%）、#11（77.16%）；（4）左肺下叶（205例）：#5、#6（88.78%）、#7（92.68%）、#9（74.15%）、#10（94.63%）、#11（84.39%），见图1。

**图1:**
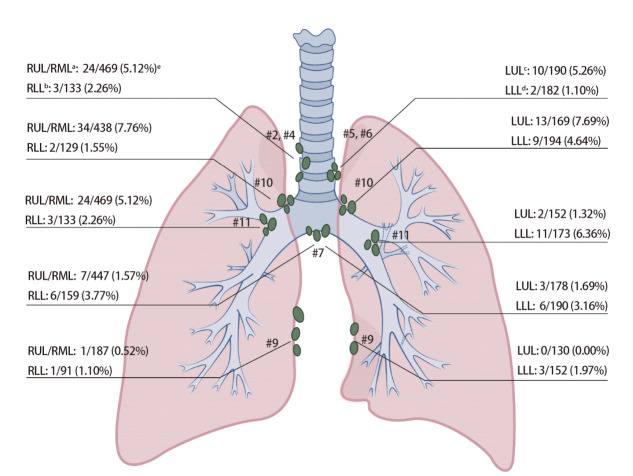
各肺叶淋巴结转移率。^a^：右肺上叶/右肺中叶；^b^：右肺下叶；^c^：左肺上叶；^d^：左肺下叶；^e^：淋巴结阳性例数/淋巴结采集例数（淋巴结转移率）。

不同部位肺叶切除术淋巴结转移率分别为：（1）右肺上叶、右肺中叶（484例）：#2R、#4R（5.12%）、#7（1.57%）、#9（0.53%）、#10（7.76%）、#11（2.49%）、#12（1.11%）；（2）右肺下叶（165例）：#2R、#4R（2.26%）、#7（3.77%）、#9（1.10%）、#10（1.55%）、#11（7.14%）、#12（3.70%）；（3）左肺上叶（197例）：#5、#6（5.26%）、#7（1.69%）、#10（7.69%）、#11（1.32%）、#12（4.26%）；（4）左肺下叶（205例）：#5、#6（1.10%）、#7（3.16%）、#9（1.97%）、#10（4.64%）、#11（6.36%）、#12（3.57%），见图1。

### 2.4 同侧不同部位（上下叶）淋巴结转移情况的χ^2^检验分析

根据NCCN指南淋巴结采集标准，即肺门淋巴结+至少三组纵隔淋巴结采样/清扫，共485例符合上述标准，其中右肺上叶+右肺中叶167例，右肺下叶71例，左肺上叶118例，左肺下叶129例。分析纵隔淋巴结转移情况得出以下结论：右肺上、中叶较右肺下叶更易出现#2R、#4R、#9淋巴结转移（P<0.05），右肺下叶则更易出现#7淋巴结转移（P<0.05）；左肺上叶较下叶更易出现#5、#6淋巴结转移（P<0.05），#7、#9转移无统计学差异（P>0.05）。见表3。

**表3:** 比较同侧不同部位的淋巴结转移情况

Lymphatic site		Right upper lobe+Right middle lobe (n=167)	Right lower lobe (n=71)	Left upper lobe (n=118)	Left lower lobe (n=129)	P
#2R, #4R	+	13	0			0.016^a^
	-	154	71			
#7	+	0	2			0.029^a^
	-	167	69			
#9	+	1	0			0.021^a^
	-	166	71			
#5, #6	+			6	1	0.041^b^
	-			112	128	
#7	+			3	3	0.912^b^
	-			115	126	
#9	+			0	2	0.174^b^
	-			118	127	

According to the protocol of at least three groups of mediastinal lymph nodes dissection recommended by National Comprehensive Cancer Network guidelines, a total of 485 patients were selected for statistical analysis.

^a^: Right upper lobe+Right middle lobe vs Right lower lobe between the same sites; ^b^: Left upper lobe vs Left lower lobe between the same sites.

### 2.5 N1、N2淋巴结转移情况的χ^2^检验分析

N1组淋巴结（#10、#11）与N2组淋巴结（#2R、#4R、#5、#6、#7、#9）转移有明显相关性（P<0.01）。见表4。

**表4:** 肺门淋巴结与纵隔淋巴结转移相关性

Lymphatic site		#2R, #4R (n)			#5, #6 (n)			#7 (n)			#9 (n)	
		+	-		+	-		+	-		+	-
#10, #11	+	12	15		7	20		11	40		2	28
	-	2	354		0	257		3	604		2	341
P		<0.01			<0.01			<0.01			<0.01	

Selecting criteria: sample mediastinal lymph nodes and at least one group of hilar lymph node (#10 or #11) at the same time.

## 3 讨论

由于肺癌筛查技术的日益成熟，越来越多的早期肺癌被发现。目前I、II期NSCLC的最佳治疗方案为手术切除+淋巴结清扫^[6]^。淋巴结的病理可以决定患者的分期、预后，掌握其转移规律有助于术者更加精准地采集淋巴结。随着人们对早期NSCLC的认知逐步深入，术中采集淋巴结的方式也在发生改变。彻底清扫纵隔淋巴结可以充分地评估淋巴转移的情况，但从解剖的角度来看，肺肿瘤细胞发生淋巴转移一般会遵循特定的路径，不同肺叶的肿瘤细胞会随着淋巴管通路转移至特定的淋巴结区域，较少发生非特异性区域淋巴结转移的情况^[9]^。

对于进展期和局部晚期的NSCLC，SND显然是更合适的术式，可以更全面地评估淋巴结的情况，有利于发现淋巴结微转移灶，但对于早期NSCLC，SND增加了乳糜胸、喉返神经损伤的概率^[10]^，过多地清扫淋巴结还会降低特异性CD8^+^细胞对肿瘤细胞的抑制作用^[11]^。ACOSOG Z0030的随机对照研究结果显示，SND并不能提高早期NSCLC淋巴结转移阴性患者的生存率^[12]^；Luo等^[13]^对5个中心的随机对照试验统计后发现SND和LSND的1、5年总生存期（overall survival, OS）及1、3、5年无病生存期（disease-free survival, DFS）均无显著差异，但LSND的3年OS优于SND（P<0.05），且SND有更多的术后并发症。Sugi等^[14]^的随机对照试验结果示对于肿瘤直径<2 cm的NSCLC，SND病死率明显高于LSND。因此越来越多的术者在探寻更小但更精准的淋巴结清扫范围来替代SND。

不同肺叶的特异性淋巴结区域尚未被完全统一。最新NCCN指南建议早期NSCLC术中应采样肺门淋巴结+至少三组纵隔淋巴结，美国外科医师学会（American College of Surgeons, ACS）认为，不应再要求淋巴结清扫数量>10枚，而应注重相关淋巴结站点的采样清扫^[15]^。Okada等^[8]^认为：上叶的肿瘤需进行上纵隔+肺门淋巴结清扫，肺门淋巴结与#7组淋巴结均为阴性的下叶肿瘤，不需要行上纵隔淋巴结清扫。Asamura等^[16]^认为：位于右上肺叶、左肺上叶固有段的肿物需清扫上纵隔淋巴结，切除右中肺叶、右下肺叶和左肺下叶的肿瘤需清扫上、下纵隔淋巴结及#7组淋巴结，切除左上舌段肿瘤需清扫左上纵隔及#7组淋巴结。Yang等^[17]^认为需要结合肿瘤直径决定淋巴结清扫区域，如肿瘤直径<1 cm，上叶肿瘤无需行下纵隔淋巴结清扫，下叶肿瘤无需行上纵隔淋巴结清扫，如肿瘤直径≤3 cm，右肺上叶肿瘤更易出现#4R转移，左肺上叶肿瘤更易出现#5转移，右肺中叶及双侧下肺更易出现#7转移。Zhang等^[18]^对cT1N0M0的患者进行了更详细的划分，他们建议：实性成分占比（consolidation tumor ratio, CTR）≤0.5或肿瘤病理为贴壁型IAC的肿瘤可以不进行纵隔淋巴结清扫；位于上叶尖段或位于上叶非尖段但无肺门阳性淋巴结及VPI的肿瘤可不进行下纵隔淋巴结清扫；位于左下叶背段且肺门淋巴结阴性的肿瘤可不进行#4L的清扫；位于左下叶基底段且肺门淋巴结阴性的肿瘤可不进行上纵隔淋巴结的清扫；其余不满足上述特征的肿瘤需进行SND。还有学者^[19]^发现当术中快速病理示#11组淋巴结阳性，或当患者满足高癌胚抗原（carcinoembryonic antigen, CEA）水平、肿瘤位于右下肺叶、#11组淋巴结阳性时，应对这些患者进行更加全面的纵隔淋巴结清扫，否则会遗漏非特异性区域阳性淋巴结的采集^[20]^。我中心数据得出的结论基本与上述学者一致，但出现了1例右肺上叶NSCLC合并#9转移的情况，这也许是部分患者右肺上叶的淋巴液经气管-食管沟回流而造成的^[21]^。

虽然越来越多的学者支持LSND可以应用于早期NSCLC的手术中，但当患者具备一些肿瘤高侵袭性的危险因素时，其发生淋巴结转移的风险会相应提高。NSCLC的组织学分型以浸润性腺癌为主，其在2011年IASLC/ATS/ERS分类中被分为五种亚型，微乳头型或实体型腺癌的侵袭性较高、预后较差，更易出现淋巴结阳性的情况^[22]^。VPI标志着肿瘤的高侵袭性^[23]^，脏层胸膜下的淋巴液回流可以不经过肺内淋巴通路，直接至上纵隔和支气管旁淋巴结，从而导致跳跃性转移，预后较差^[21,24]^。STAS、脉管侵犯也是淋巴结转移的相关因素，虽然这无法在术中快速病理中体现，但术后的常规病理中，如发现肿瘤细胞经气道播散至主病灶以外的肺实质中，或沿着微血管、微淋巴管移行，通常预示着患者预后较差。肿瘤的直径也与淋巴结转移相关，直径越大，淋巴结转移风险越高^[25]^，还有学者^[26]^提出隐匿性淋巴结转移的概念，并认为对于cT1N0的患者，肿瘤直径每增加1 cm，其隐匿性转移风险就会增加3倍。因此对于合并上述高危因素的患者，我们应重视其较高的淋巴结转移风险，在围手术期为其选择更为全面的治疗方案。

本研究也有不足之处。首先这是一项回顾性研究，术者对不同部位的肿瘤有不同的淋巴结采样侧重；其次，如患者存在年龄较大、术前肺功能较差、术中胸腔黏连较重等影响术中采集淋巴结的因素，术者会主动地减少纵隔淋巴结的采集，降低术中风险，减少术后并发症的发生；病例缺少影像学特征统计，对于CT影像上CTR更多、肺结节直径越大的病例，术者会主动增加淋巴结站数、淋巴结个数的清扫，这些主观因素可能会对研究结果产生影响；最后，我们缺少对术后病理证实为淋巴结转移的患者进行生存分析，并未能够证实LSND对此类患者是否具有可行性。

总之，由于SND可能使无淋巴结转移的肿瘤直径≤2 cm的早期NSCLC患者术中、术后并发症发生率上升，我中心建议对早期NSCLC患者行手术治疗时，可选择LSND，其中右肺上叶、右肺中叶采样#2R、#4R、#7；右肺下叶采样#2R、#4R、#7、#9；左肺上叶、左肺下叶采样#5、#6、#7、#9。我们筛选清扫符合NCCN指南标准的病例后比较，发现右肺上叶、右肺中叶的肿瘤更易出现#2R、#4R、#9的转移，右肺下叶更易出现#7的转移，左肺上叶更易出现#5、#6的转移，且当N1淋巴结阳性时，上、下纵隔淋巴结均应进行采样/清扫。同时我们发现，当患者满足性别为男性、病理为非腺癌、肿瘤直径大于1 cm但不大于2 cm、存在STAS、VPI、有脉管浸润、腺癌低分化、腺癌病理亚型为微乳头型或实体型时，更易发生淋巴结转移，需进一步完善此类患者预后数据，给出淋巴结采样/清扫的建议。

**Competing interests**

The authors declare that they have no competing interests.

**Author contributions**

Chen L, Wu WB and He ZC conceived and designed the study. Jin TY and Li ZH analyzed the data. Jin TY, Tang JW and Xu J contributed analysis tools. Chen L and He ZC provided critical inputs on design, analysis and interpretation of the study. All the authors had access to the data. All authors read and approved the final manuscript as submitted.
